# Back to the future: a new ‘old’ lead for tuberculosis

**DOI:** 10.1002/emmm.201201811

**Published:** 2012-09-17

**Authors:** Gerard D Wright

**Affiliations:** Department of Biochemistry and Biomedical Sciences, M.G. DeGroote Institute for Infectious Disease Research, McMaster UniversityHamilton, Ontario, Canada

**Keywords:** antibiotic, InhA, pyridomycin, resistance, tuberculosis

See related article in EMBO Molecular Medicine http://dx.doi.org/10.1002/emmm.201201689

‘The Great White Plague, which only 10 years ago was thought to be immune to drug therapy, is gradually being eliminated… Streptomycin pointed a way. Later supplemented with PAS (para-amino salicylic acid) and more recently with isoniazid, it has brought the control of this disease within sight’. Selman A. Waksman, Noble Prize Banquet Speech 1952.

Despite the breakthroughs in management of tuberculosis noted by Waksman 8 years after his discovery of the first effective antitubercular drug, streptomycin (Schatz et al, 1944), the disease continues to infect, incapacitate and kill in the 21st century. Over 8.8 million are infected and 1.4 million die every year (http://www.who.int/mediacentre/factsheets/fs104/en/index.html). The organism that causes tuberculosis, *Mycobacterium tuberculosis*, has proven to be a formidable match for our ability to control disease. At the core of this challenge is the remarkable physiology and biochemistry of *M. tuberculosis* and its ability to reside in a dormant state within human macrophages. These unique characteristics necessitate multidrug therapy, typically ≥4 antibiotics, and extended periods of dosing (6–12 months). The rise of strains resistant to multiple drugs MDR (multi-drug-resistant) resistant to rifampin and isoniazid, XDR (extensive-drug-resistant) resistant to even more antibiotics, and now some strains totally impervious to all available drugs, TDR (totally drug-resistant) raises the specter of the emergence of a global health care disaster and a return to a pre-Waksman era of tuberculosis treatment (Zumla et al, 2012). The result is a great need for new therapies and a renewed effort to find new anti-TB antibiotics.

» The result is a great need for new therapies and a renewed effort to find new anti-TB antibiotics. «

**Figure 1:**
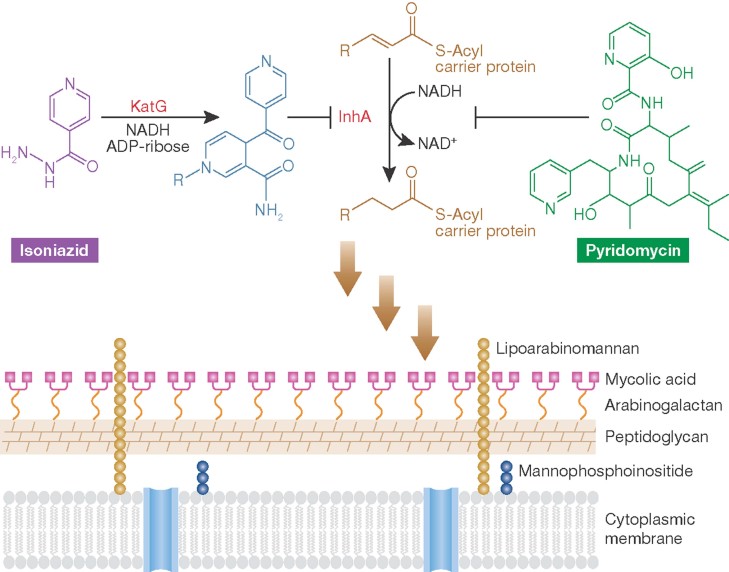
Inhibition of mycolic acid synthesis by isoniazid and pyridomycin Pyridomycin inhibits the enoyl acyl carrier protein reductase InhA, which is essential for the formation of the mycolic acids component of the cell wall of *Mycobacterium tuberculosis*. InhA is also the target for the anti-tubercular drug isoniazid, which requires activation by KatG.

While the natural product streptomycin ushered in antibiotic treatment for tuberculosis, the development of resistance during therapy propelled the discovery of alternative drugs including the synthetic compound isoniazid in the early 1950s. Isoniazid blocks the synthesis of mycolic acids that are key protective components of the *M. tuberculosis* cell wall by inhibiting the essential fatty acid synthesis enzyme enoyl-acyl-carrier protein reductase, InhA (Fig 1). Isoniazid is a pro-drug that must be intracellularlymetabolized by *M. tuberculosis* to its active form where it can generate an adduct with NADH that is a potent (sub nM *K*_i_), slow, tight-binding inhibitor of InhA (Rawat et al, 2003; Rozwarski et al, 1998). This remarkable domino effect mechanism of action highlights the importance and vulnerability of the mycobacterial cell wall, which is also the target of a second highly effective synthetic antitubercular drug ethambutol. However, despite the dominance of natural product antibiotics for the treatment of most other bacterial infections and also as front line anti-tubercular drugs (*e.g.* rifampin), there have been no lead natural products that target mycobacterial cell wall synthesis.

In this issue of EMBO Molecular Medicine, Hartkoorn et al (2012) report the mechanism of action of an ‘old’ anti-tubercular natural product, pyridomycin, first reported in 1953. This compound was identified in extracts of the soil microbe *Streptomyces pyridomyceticus* and more recently from *Dactylosporangium fulvum*. Pyridomycin (Fig 1) is a hybrid polyketide-peptide with excellent anti-mycobacterial activity without off target effects on other bacteria, including related genera such as *Corynebacteria*, suggesting that its use will not select for resistance in other bacteria. Importantly, the authors find that pyridomycin is bactericidal against *M. tuberculosis*, has activity against the bacteria growing within macrophages and shows low toxicity to human cells and mice. This excellent spectrum of activity is reminiscent of the best anti-mycobacterial drugs including cell wall active agents such as isoniazid.

To identify the cellular target of pyridomycin, Hartkoorn et al selected resistant mutants by growth on pyridomycin followed by whole genome sequencing. This identified a single mutation in the *inhA* gene that converts Asp148 to a Gly residue. Follow-up genetic studies were consistent with identification of InhA as the target for pyridomycin, the same target for the highly successful synthetic drug isoniazid. Furthermore, like isoniazid, the activity of pyridomycin reduces mycolic acid production and impairs the mycobacterial cell wall. Because isoniazid is a prodrug that requires metabolic activation by the catalase–peroxidase KatG, drug resistance is most frequently associated with mutations in *katG*. Importantly isoniazid-resistant clinical isolates impaired in *katG* activity retained sensitivity to pyridomycin, demonstrating that this natural product has a different mode of inhibition of InhA and offering an orthogonal point of target inhibition. The authors then went on to detailed studies of the enzymology of inhibition to reveal that pyridomycin is a competitive inhibitor of NADH at the InhA activity site, a new mode of action for drug intervention that is distinct from the isoniazid-NADH adduct. This is a great example of the multidisciplinary approach that is needed to identify and characterize new antibiotic drugs leads that combines clinical microbiology, molecular knowledge of resistance, chemistry, genomics and fundamental protein chemistry to pinpoint mode of action and rationalize bioactivity.

» The identification of a natural product inhibitor of InhA with antimycobacterial effects is an important breakthrough. «

The identification of a natural product inhibitor of InhA with antimycobacterial effects is an important breakthrough. Other synthetic compounds such as triclosan and ethionamide also target InhA, the later through a prodrug activation mechanism similar to isoniazid; however, these compounds have marginal effects on therapy and in the case of triclosan, no clinical utility for tuberculosis treatment. Nevertheless, these compounds point to the critical role that InhA and mycolic acid biosynthesis has on mycobacterial physiology and as a drug target. The identification of pyridomycin as a new bioactive inhibitor of InhA, with a new mode of action that is impervious to clinical isoniazid-resistant strains is a great leap forward in identifying a new chemical scaffold able to penetrate both mammalian and *M. tuberculosis* cells to kill pathogens. This finding offers a new lead molecule for new further antimycobacterial drug development.

This work also points out the importance of reinvestigating abandoned molecules as leads for new antibacterial agents. There is a tremendous need for new antibiotics, yet there is a real innovation gap in identifying new chemical scaffolds that can be championed as leads in drug discovery programs (Fischbach & Walsh, 2009; Wright, 2012). The result is a growing clinical crisis and an increasingly disengaged drug discovery sector (Cooper & Shlaes, 2011). Revisiting ‘old’ or abandoned antimicrobial scaffolds to identify new targets and thereby rejuvenate medicinal chemistry campaigns offers a creative approach to bridging the antibiotic innovation gap. It has been estimated that 25,000–30,000 natural product antibiotics have already been identified over the past 70 years (Berdy, 2005). This provides a remarkably rich pool of bioactive compounds that have the potential to act as probes to identify new targets and importantly also to serve as chemical leads for new drugs. The revisiting of the lipopeptideantibiotic daptomycin, abandoned in the 1980s but resurrected a decade later, offers proof of the success of such a strategy (Baltz et al, 2005). A dosing strategy for this natural product that differs from the original approach of the 1980s has resulted in a powerful drug for the treatment of drug-resistant Gram-positive pathogens and annual sales that approach $1 billion. The work of Hartkoorn et al reported here demonstrates that a similar return to the past offers new hope to identify leads for desperately needed anti-tuberculosis drugs. These examples offer a way forward in antibiotic drug discovery; a field that is anxiously searching for new paradigms for success. ‘Old’ antibiotics are worthy of a renewed look with fresh eyes and 21st century to meet the antibiotic crisis.
